# Monoclonal Gammopathy of Clinical Significance-Associated Glycogen Storage Myopathy: A Novel Acquired Muscle Disease

**DOI:** 10.7759/cureus.91393

**Published:** 2025-09-01

**Authors:** Mohamed R Belkhribchia, Tarik Toua, Johannes A Lobrinus, Jean-Michel Vallat, Jaouad Salmaoui

**Affiliations:** 1 Department of Neurology, Hassan II Regional Hospital, Dakhla, MAR; 2 Department of General Medicine, Hassan II Regional Hospital, Dakhla, MAR; 3 Department of Pathology, Geneva University Hospitals, Geneva, CHE; 4 Department of Neurology, University Hospital of Limoges, Reference Center for Rare Peripheral Neuropathies, Limoges, FRA

**Keywords:** chemotherapy, dexamethasone., electron microscopy, glycogen storage myopathy, melphalan, monoclonal gammopathy of clinical significance

## Abstract

Monoclonal gammopathy of clinical significance (MGCS)-associated myopathy is a category of diseases in which the clonal plasma cells are responsible for muscle damage. MGCS-associated myopathy includes amyloid light chain (AL) amyloidosis-associated myopathy, sporadic late-onset nemaline myopathy with monoclonal gammopathy (SLONM-MG), and non-amyloid light chain deposition disease (LCDD)-associated myopathy. On the other hand, glycogen storage myopathy (GSM) is a well-known genetic condition affecting the glycogen biosynthesis or degradation pathways. Nevertheless, in exceedingly rare situations, GSM can be acquired and associated with MGCS.

Recently, the acquired MGCS-associated GSM was recognized within the category of MGCS-associated myopathy. Herein, we report the case of a 62-year-old male patient who developed an MGCS-associated GSM. The weakness was subacute and affected axial, proximal, and distal muscles. An obvious asymmetry also characterized the clinical presentation of this myopathy, and serum creatine kinase was normal. The patient responded significantly to chemotherapy based on a protocol of eight cycles of melphalan-dexamethasone. Due to the treatability of MGCS-associated GSM, it is of utmost importance to differentiate this exceptional and acquired myopathy from the usual genetic GSM.

## Introduction

Monoclonal gammopathies of clinical significance (MGCS) is a term employed to describe monoclonal gammopathies that cause diseases with clinical manifestations. Unlike monoclonal gammopathies of undetermined significance (MGUS), the proliferation of the clonal plasma cells in MGCS is responsible for organ damage and its related myriad of clinical symptoms and signs. Of note, monoclonal gammopathies of renal significance (MGRS) are the best known among the category of MGCS [1]. 

In the same context, MGCS-associated myopathy is a group of rare disorders in which the clonal plasma cells are responsible for muscle damage. The latter group includes amyloid light chain (AL) amyloidosis-associated myopathy, sporadic late-onset nemaline myopathy with monoclonal gammopathy (SLONM-MG), and the exceptional non-amyloid light chain deposition disease (LCDD)-associated myopathy [1-6]. 

More recently, a novel acquired disease within MGCS-associated myopathy was recognized and is defined by the presence of an accumulation of glycogen in the muscle in association with MGCS. This acquired glycogen storage myopathy (GSM) was reported to respond to immunotherapy and/or chemotherapy [7-9]. 

Herein, we report the case of a 62-year-old male patient who developed MGCS-associated GSM. A therapeutic protocol combining melphalan and dexamethasone (MDex) allowed a significant clinical and hematological response in our patient. 

## Case presentation

A 62-year-old man suffered from a painless weakness of the four limbs, which had progressed during the preceding six months. The weakness predominated in the left lower limb (LL) and the right upper limb (UL). The weakness had a rapidly progressive evolution, and the patient became bedridden and no longer able to stand and walk after six months from the onset of the weakness. The passage from lying to the sitting position also became impossible. Apart from diabetes type 2 managed by insulin, his personal and familial medical history was negative for hereditary neuromuscular diseases. 

The physical examination revealed proximal weakness in the UL, more marked on the right side (3/5 according to the Medical Research Council (MRC) score) than the left side (4/5). The distal parts of the UL were also affected by the weakness at 4/5 on both sides. The weakness was marked on the left LL and evaluated at 1/5 on both proximal and distal muscles. The right LL showed a less severe weakness with 3/5 in the proximal muscles, whereas its distal muscles were normal (5/5). The cervical muscles were weak with 3/5 in the flexors and extensors of the neck.The Achilles reflex was abolished bilaterally in addition to hypoesthesia on feet. The ocular and facial muscles were unremarkable, and the patient had no dysphagia, no dysarthria, nor dyspnea. 

Electromyography (EMG) test demonstrated a myopathic pattern in weak, proximal and distal muscles. Spontaneous activity such as fibrillations potentials (FP) and positive sharp waves (PSW) were present in some of these muscles. A diminution of the amplitudes of the sensory nerve action potentials (SNAP) of the sural nerves was also present indicating a coexisting distal axonal sensory polyneuropathy. No anomalies supporting a plexopathy or a radiculopathy were revealed on EMG. The serum creatine kinase (CK) was within normal range. The transthoracic echocardiography and electrocardiogram were unremarkable. At this stage, the diagnosis of a rapidly progressive myopathy was made. Additional diabetic, length-dependent, sensory and axonal polyneuropathy was also noted. 

The panel of the antibodies related to auto-immune myopathies was unremarkable. The auto- antibodies anti-SSA, anti-SSB, hepatitis B, C and HIV serologies were negative. The hematologic, metabolic and hormonal analysis such as complete blood count, vitamin D, calcemia, phosphoremia, creatinine, urea, aminotransferases and TSH were normal. Nevertheless,the serum protein electrophoresis (SPE) disclosed a spike in the beta-globulins at the concentration of 6,7 g/L. The serum immunofixation (IF) confirmed the presence of IgA kappa monoclonal immunoglobulin. The serum free light chains (FLC) test revealed increased kappa light chains at 55,16 mg/L (N: 3,30 to 19,40) with normal lambda light chains at 15,90 mg/L (N: 5,71 to 26,30). The kappa/lambda ratio was elevated at 3,47 (N: 0,26 to 1,65) confirming the presence of monoclonal kappa light chains in the serum. The Bence-Jones proteinuria was negative, and the bone marrow biopsy was normal.The skeletal imagery was unremarkable. 

The late age at onset, the negative personal and familial history for myopathy, the rapidly progressive evolution and the association with MG led us to prioritize an acquired disease, particularly a MGCS-associated myopathy. Hence, the main hypothesis at this stage were AL amyloidosis-associated myopathy and SLONM-MG. 

The first muscle biopsy (right deltoid muscle) showed rare atrophic muscle fibres, scarce abnormal megamitochondria with paracrystalline inclusions. Some subsarcolemmal membrane vacuoles with autophagic material and cellular debris were observed. Nevertheless, all the following pathological changes were absent: nemaline bodies, amyloid deposits, glycogen accumulation, inflammatory infiltrate and vasculitis. 

**Figure 1:**
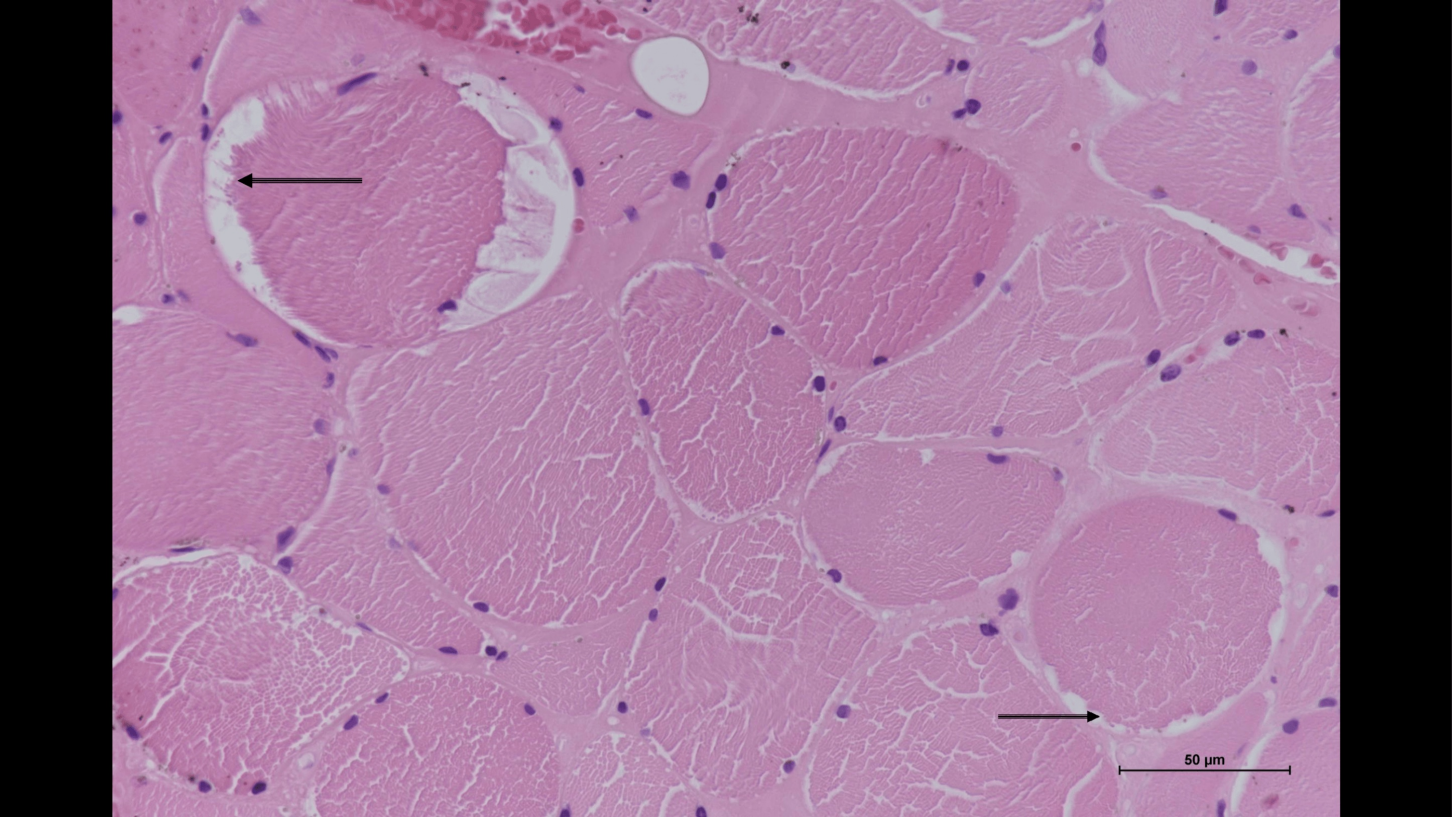
Left tensor fascia lata muscle biopsy Transverse paraffin sections (Hematoxylin and Eosin stain): Presence of significant atrophic myofibres. No necrotic, regenerating fibres or inflammatory cell  infiltrate were observed. Few vacuoles, mainly subsarcolemmal (arrows), are seen; most are optically empty because glycogen was dissolved during processing (thus, periodic acid Schiff staining was not performed). A mild increase in endomysial connective tissue is observed.

**Figure 2:**
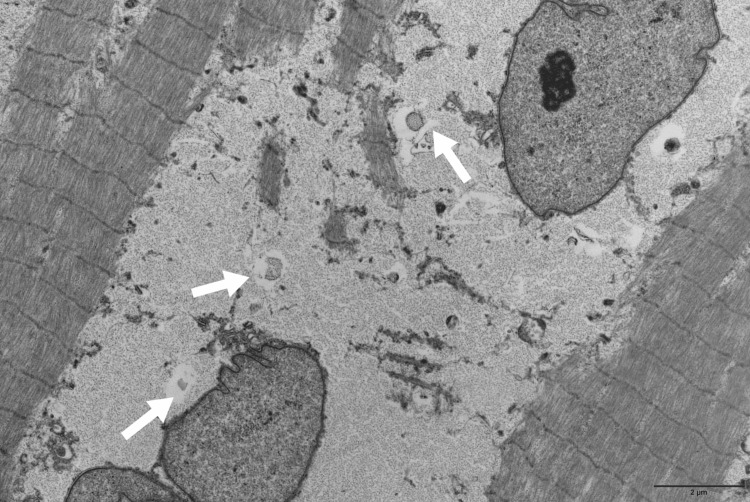
Left tensor fascia lata muscle biopsy Electron microscopy, longitudinal section (Thiéry staining): Intra-sarcoplasmic accumulation of glycogen (arrows) between myofibrils.

**Figure 3:**
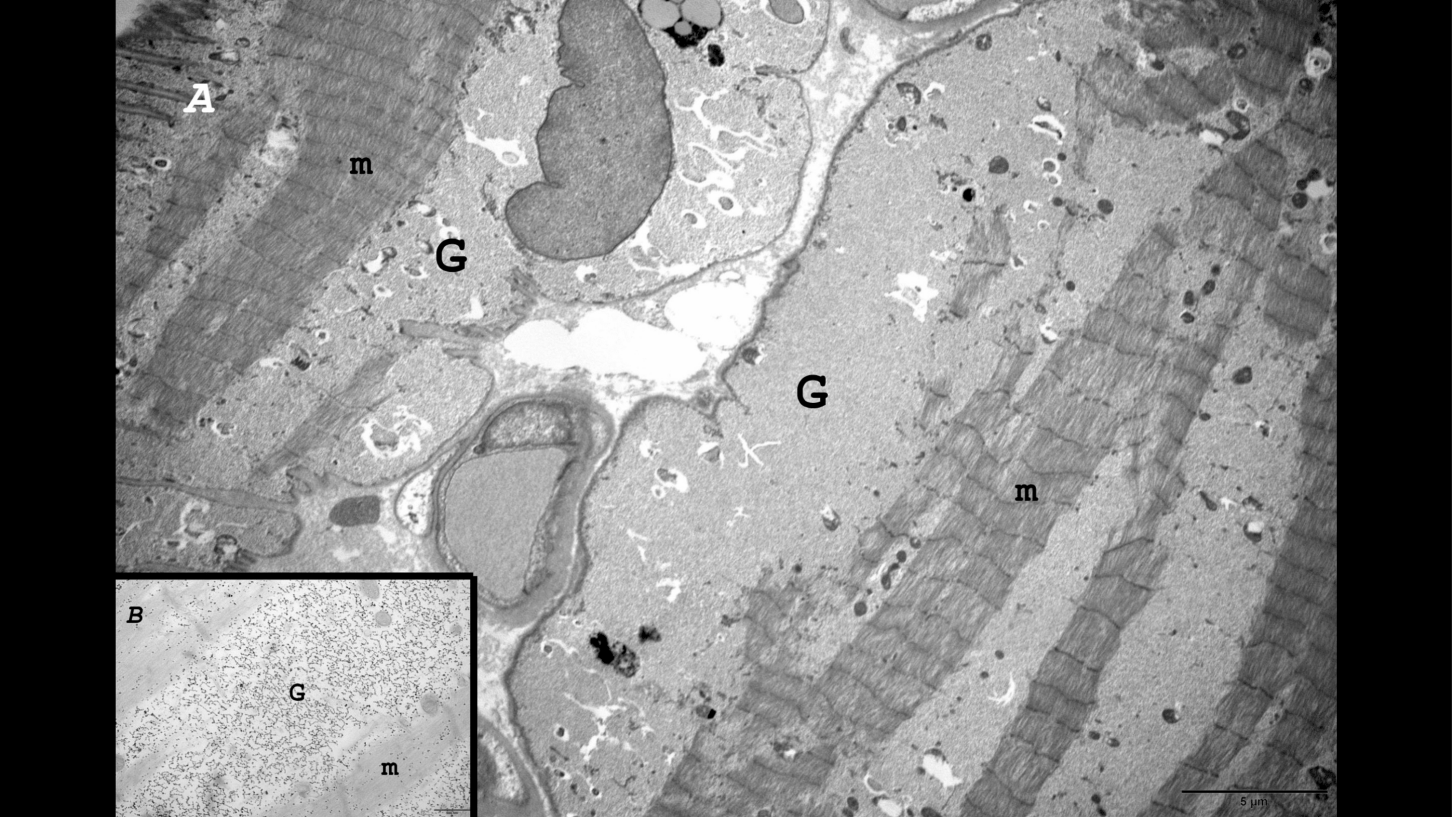
Left tensor fascia lata muscle biopsy Electron microscopy, longitudinal section (Thiéry staining): A) In two muscular cells, diffuse extralysosomal intra-sarcoplasmic accumulation of glycogen which predominates in the subsarcolemmal areas (G); m: myofibrils. B) Glycogen granules are specifically black stained by the Thiéry technique.

**Figure 4:**
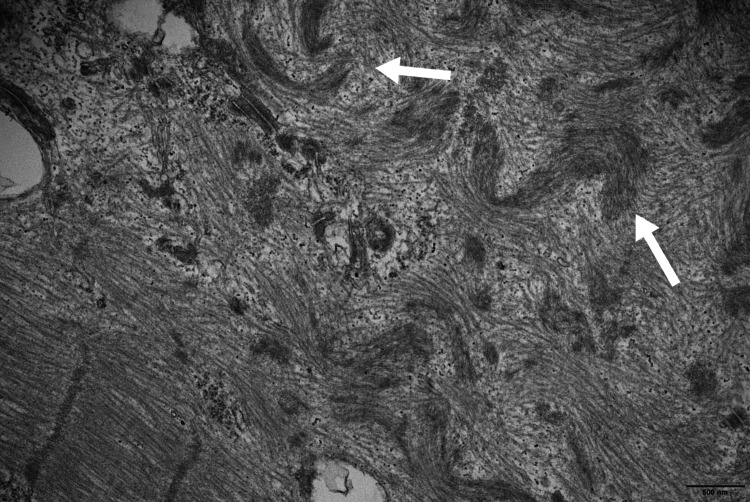
Left tensor fascia lata muscle biopsy Electron microscopy, longitudinal section (uranyl acetate and lead citrate stains): Large focal area of myofibrillar disintegration (arrows).

**Figure 5:**
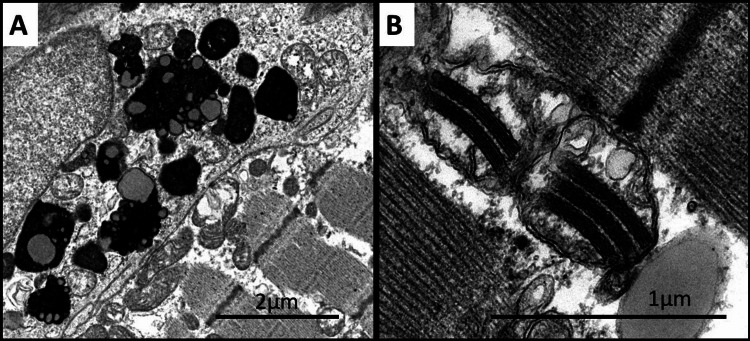
Right deltoid muscle biopsy, electron microscopy (uranyl acetate and lead citrate stains) A) shows subsarcolemmal membrane vacuolization with autophagic material and cellular debris. B) shows enlarged mitochondria with paracrystalline inclusions.

We repeated a second muscle biopsy three months later in a weaker muscle (left tensor fascia lata). This biopsy revealed severe myopathy with important variation of the diameter between the myofibres, frequent nuclear centralizations, significant proliferation of connective and adipose tissue within the interstitium and some subsarcolemmal membrane vacuolization. Notably, a massive accumulation of glycogen within the sarcoplasms of numerous myofibres was demonstrated by electron microscope. The glycogen was located essentially under the sarcolemma and between the myofibrils. The ultrastructural study also showed some focal areas of myofibrillar disintegration.No nemaline rods, amyloid deposits or inflammatory infiltrates were demonstrated in the muscle biopsy (Figures 1-5). 

Further investigations included next-generation sequencing of GSM-related genes and was negative. Therefore, we made the final diagnosis of an acquired MGCS-associated GSM. 

As our patient dismissed the option of receiving an autologous stem cell transplant (ASCT), we started a chemotherapy protocol (MDex) aimed at eradicating the MGCS. Due to limited financial resources, the patient could not afford newer chemotherapeutic agents such as lenalidomide or bortezomib. In parallel, a program of motor rehabilitation was continued. MDex protocol consisted of oral melphalan (10 mg/m2 body surface area) plus oral dexamethasone (40 mg/d) on days one to four of every 28-day cycle. Prophylaxis with proton- pump inhibitor was administered on days one to five. A total of nine cycles were scheduled. Due to the presence of indicators of poorly controlled diabetes (elevated glycated hemoglobin and axonal sensory polyneuropathy), an adjustment to the diet and insulin therapy was made. 

After seven cycles of MDex, the passage from the lying to the sitting position, without assistance, became possible. The patient became also able to walk with crutches for several minutes daily (approximately 15 minutes per day). The autonomy was regained in daily activities such as bathing and feeding. At this time, the strength significantly improved: the weakness of the proximal parts of the right UL, LL and left UL fully resolved. The weakness in the distal parts of the UL disappeared whereas the weakness of the proximal and distal parts of the left LL significantly improved from 1/5 to 4/5. 

The hematological tests (after seven cycles of Mdex) revealed the persistence of only two unquantifiable faint monoclonal bands IgA and IgG both of kappa type. The free kappa chains decreased to 12.23 mg/L, and the kappa/lambda ratio normalized to 1.34. Notably, the protocol of MDex was well tolerated without any significant side effect. Due to the significant hematological and clinical response, we reduced the number of the cycles of MDex to 8 rather than the initial planned nine cycles. One year after the initiation of the chemotherapy, the residual weakness of the left LL resolved. A long-term and regular follow-up was scheduled to diagnose any eventual relapse.

## Discussion

MGCS-associated myopathy is a rare group of diseases in which the MG causes significant muscle damage. AL amyloidosis-associated myopathy is the most well-known disease within MGCS-associated myopathy. SLONM-MG and other rare conditions like non-amyloid LCDD-associated myopathy also belong to this category of MGCS-associated myopathy [1-6].

On the other hand, GSM are hereditary disorders of the glycogen biosynthesis or degradation pathways. Patients may present with rhabdomyolysis or progressive limb weakness affecting predominantly the proximal or distal musculature [10].

Notwithstanding, in exceedingly rare settings, GSM can be the expression of MGCS-associated myopathy and thus represents an acquired myopathy. Acquired GSMs belonging to the category of MGCS-associated myopathy are exceptional and were recently reported only by a few authors (Table 1). 

**Table 1:** The epidemiological, clinical and paraclinical features with therapeutics and outcomes in the published cases of MGCS-associated GSM MGCS - monoclonal gammopathy of clinical significance; GSM - glycogen storage myopathy; M - male; F - female; UL - upper limbs; LL - lower limbs; RS - right side; LS - left side; POEMS - polyneuropathy, organomegaly, endocrinopathy, M-protein and skin changes; CK - creatine kinase; IU/L - international unit/liter; EMG - electromyography; FP - fibrillation potentials; PSW - positive sharp waves; CRD - complex repetitive discharges; MG - monoclonal gammopathy; FLC - free light chain; MDEX - melphalan-dexamethasone; ASCT - autologous stem cell transplant

	Present case	Allenbach et al., 2019 [7]	Soontrapa et al., 2023 [8]
Age at onset (years)	62	37, 46, 56	51
Sex	M	2F, 1M	F
Clinical features	Subacute weakness of axial, proximal, and distal muscles, LL >UL, asymmetry (LS>RS), painless, and lack of systemic manifestations.	Subacute, symmetrical weakness of axial and proximal muscles, marked stiffness, severe weight loss, dysphagia, dysarthria (in two patients), no systemic manifestations.	Context of POEMS syndrome (foot drop, LL edema, weight loss, severe distal weakness, length-dependent sensory deficits), myopathy: mild proximal weakness.
Serum CK (IU/L)	Normal	Increased: 1400, 1600, 2974	Normal
EMG	Abnormal spontaneous activity (FP, PSW), myopathic pattern, additional sensory axonal polyneuropathy in LL.	Abnormal spontaneous activity: abundant CRD, myopathic pattern.	Severe mixed demyelinating and axonal sensorimotor polyradiculoneuropathy (POEMS syndrome), proximal myopathic pattern + FP.
Muscle biopsy findings	Severe myopathic lesions, massive accumulation of free glycogen, autophagic material, mitochondrial abnormalities, myofibrillar disintegration.	Vacuoles containing glycogen, autophagic material, sarcolemma complement membrane attack complex deposits.	Scattered fibers with glycogen-filled vacuoles, rare fibres: polyglucosan bodies.
MG	Ig A Kappa, monoclonal FLC Kappa (in serum)	Ig G Kappa (in all patients)	Biclonal gammopathy (IgG Kappa, Ig A lambda)
Genetic analysis	Negative	Negative	Negative
Therapeutics	Chemotherapy: 8 MDex cycles	Combination of corticosteroids, intravenous immunoglobulins, and immunosuppressants.	ASCT
Evolution after treatment	Walking ability regained, hematological response: normalization of the serum FLC ratio and the persistence of two unquantifiable monoclonal bands of kappa type.	Walking ability recovered (in 2 patients), MG: very low level or undetectable.	Walking ability regained

In 2019, the first three cases of MGCS-associated GSM were described by Allenbach et al. [7]. Then, the same cases were prospectively studied and reported in a recent report [9]. These three patients developed a subacute myopathy in addition to marked stiffness. The most relevant pathologic finding was the presence of vacuoles filled with glycogen in the muscle biopsies. These cases all had MG and responded to intravenous immunoglobulin (IVIG) and immunosuppressive therapies. The authors termed this MG-associated GSM as vacuolar myopathy with monoclonal gammopathy and stiffness (VAMMGS) [7,9].

In 2023, in the same setting, Soontrapa et al. reported the case of a female patient with polyneuropathy, organomegaly, endocrinopathy, M protein, and skin changes (POEMS) syndrome. The patient also developed a mild proximal myopathy. The muscle biopsy demonstrated fibres with glycogen-filled vacuoles with additional rare myofibres containing polyglucosan bodies. The patient had a biclonal gammopathy, and the myopathy improved after the ASCT [8].

In our case, the patient developed a rapidly progressive, painless weakness of all limbs. The topographic pattern of the weakness was asymmetric and involved both the proximal and distal muscles of the limbs. The axial muscles were also severely affected. Unlike the VAMMGS, our patient did not develop stiffness, and the serum CK was within normal range. The EMG revealed a myopathic pattern in the affected muscles with abnormal spontaneous activity (FP, PSW). IgA kappa MG and free monoclonal kappa chains were revealed in the serum. Muscle biopsy showed a severe GSM. There was no evidence for hereditary glycogen metabolic disorder throughout genetic studies. The protocol of MDex has led to a significant hematological response in parallel to the neurological improvement in our patient. 

Notably, several features of the MGCS-associated GSM in our patient differ from the cases reported by the previously cited authors. The older age at onset, the asymmetric topography of the weakness, and the severe involvement of the distal muscles, in addition to the normal serum CK in our case, may mimic the presentation of other neuromuscular diseases such as inclusion body myositis (IBM) or motor neuron disease (MND). Additionally, the type of abnormal spontaneous activity in EMG, which was FP and PSW in our patient, differs from the CRD in the cases of Allenbach et al. The muscle biopsy in our patient also showed some distinctive characteristics. Indeed, in addition to the accumulation of glycogen, ultrastructural alterations were also marked, such as mitochondrial abnormalities and myofibrillar disintegration. Similarly, these ultrastructural changes are also common in SLONM-MG, another MGCS-associated myopathy. 

In the continuum of MGCS-associated GSM, de Berny et al. recently reported a case of vacuolar myopathy associated with lambda light chain myeloma. Systemic involvement (renal and cardiac), empty vacuoles with lambda chain deposits in muscle, in the context of multiple myeloma, were the distinctive features of the report of de Berny et al. Therapy with chemotherapy allowed a good hematological response in parallel with significant improvement of muscular strength in this case [11].

The gold standard therapy in MGCS-associated myopathy, such as AL amyloid myopathy and SLONM-MG, is chemotherapy using ASCT and /or chemotherapeutic agents [12,13]. Hence, we used a similar approach in our case. Due to limited financial resources, the patient could not afford newer chemotherapeutic drugs, and the protocol of MDex was finally approved. The substantial clinical improvement in our patient was observed in parallel with the almost eradication of the monoclonal protein. The protocol was also well tolerated by our patient. 

## Conclusions

Relying on the present case and the previous reports, we assume that the features (clinical, biological, and electrophysiological) of these acquired MGCS-associated GSM can be variable. More cases are needed in the future to better elucidate this novel muscle disease belonging to MGCS-associated myopathy. Interestingly, its differentiation from some mimics, such as IBM and MND, and the genetic GSM is of utmost importance due to its treatability. 

## References

[1] Yu H, He D, Zhang Q, Cao B, Liu W, Wu Y (2022). Case report: Monoclonal gammopathies of clinical significance-associated myopathy: a case-based review. Front Oncol.

[2] Belkhribchia MR, Moukhlis S, Bentaoune T, Chourkani N, Zaidani M, Karkouri M (2020). Skeletal myopathy as the initial manifestation of light chain multiple myeloma. Eur J Case Rep Intern Med.

[3] Ostrow LW, Corse AM, Morrison BM, Huff CA, Carrino JA, Hoke A, Mammen AL (2012). Expanding the spectrum of monoclonal light chain deposition disease in muscle. Muscle Nerve.

[4] Kasahara N, Tamura H, Matsumura O (1994). An autopsy case of light chain deposition disease. Intern Med.

[5] Chahin N, Selcen D, Engel AG (2005). Sporadic late onset nemaline myopathy. Neurology.

[6] Naddaf E, Milone M, Kansagra A, Buadi F, Kourelis T (2019). Sporadic late-onset nemaline myopathy: clinical spectrum, survival, and treatment outcomes. Neurology.

[7] Allenbach Y, Salort-Campana E, Malfatti E (2019). P.09Vacuolar myopathy with monoclonal gammopathy and stiffness (VAMGS). Neuromuscul Disord.

[8] Soontrapa P, Tracy JA, Gonsalves WI, Liewluck T (2023). Treatment-responsive glycogen storage myopathy in a patient with POEMS syndrome: a new monoclonal gammopathy-associated myopathy. Eur J Neurol.

[9] Staedler K, Allenbach Y, Salort-Campana E (2025). Vacuolar myopathy with monoclonal gammopathy and stiffness (VAMMGAS). Eur J Neurol.

[10] Tarnopolsky MA (2018). Myopathies related to glycogen metabolism disorders. Neurotherapeutics.

[11] de Berny Q, Salle V, Renard C (2025). Vacuolar myopathy associated with lambda light chain myeloma: a case report and review of the literature. BMC Musculoskelet Disord.

[12] Kotchetkov R, Susman D, Bhutani D, Broch K, Dispenzieri A, Buadi FK (2021). Chemotherapy-based approach is the preferred treatment for sporadic late-onset nemaline myopathy with a monoclonal protein. Int J Cancer.

[13] Belkhribchia MR, Tazi I, Louhab N, Kissani N, Mahmal L, Pereon Y (2017). Autologous stem cell transplantation in a patient with sporadic late-onset nemaline myopathy and monoclonal gammopathy: first Moroccan experience. Presse Med.

